# Co-infections of respiratory pathogens and gastrointestinal parasites in smallholder pig production systems in Uganda

**DOI:** 10.1007/s00436-023-07797-4

**Published:** 2023-02-22

**Authors:** Peter Oba, Barbara Wieland, Frank N. Mwiine, Joseph Erume, Michel M. Dione

**Affiliations:** 1 International Livestock Research Institute, P. O. Box 24384, Kampala, Uganda; 2 National Agricultural Research Organization, Abi Zonal Agricultural Research and Development Institute (Abi ZARDI), P. O. Box 219, Arua, Uganda; 3 Institute for Virology and Immunology, Mittelhäusern, Switzerland; 4 Department of Infectious Diseases and Pathobiology (DIP), Vetsuisse Faculty, University of Bern, Bern, Switzerland; 5 College of Veterinary Medicine, Animal Resources and Biosecurity, Makerere University, P. O. Box 7062, Kampala, Uganda; 6 International Livestock Research Institute, c/o Africa Rice Sahel Station, Dakar, Senegal

**Keywords:** Co-infections, Helminths, Pigs, Pathogens, Respiratory, Uganda

## Abstract

A cross-sectional study was conducted to identify factors for infections of pigs with key respiratory pathogens: porcine circovirus type 2 (PCV2), porcine reproductive and respiratory syndrome virus (PPRSv), *Mycoplasma hyopneumoniae* (*M. hyo*), *Actinobacillus pleuropneumoniae* (*App*), and gastrointestinal (GI) parasites in Uganda. A structured questionnaire was used to collect data on management practices associated with infections. Ninety (90) farms and 259 pigs were sampled. Sera were screened against 4 pathogens using commercial ELISA tests. The Baerman’s method was used to identify parasite species in faecal samples. Logistic regression was done to identify risk factors for infections. Results showed individual animal seroprevalence of PCV2 was 6.9% (95% CI 3.7–11.1), PRRSv 13.8% (95% CI 8.8–19.6), *M. hyo* 6.4% (95% CI 3.5–10.5), and *App* 30.4% (95% CI 24.8–36.5). The prevalence of *Ascaris spp.* was 12.7% (95% CI 8.6–16.8), *Strongyles spp* was 16.2% (95% CI 11.7–20.7), and *Eimeria spp.* was 56.4% (95% CI 50.3–62.4). Pigs infested with *Ascaris spp.* were more likely to test positive to PCV2, odds ratio (OR) 1.86 (CI 1.31–2.60; *p* = 0.0002). For *M. hyo*, infection with *Strongyles spp.* was a risk factor (OR 12.9, *p* < 0.001). Pigs that had *Strongyles* and *Ascaris spp.* Infections (ORs 3.5 and 3.4, *p* < 0.001 respectively) were likely to have co-infections. The model showed that use of cement, elevated floor, and limiting contacts with outside pigs were protective while using mud and helminth infestations increased risks of co-infections. This study provided evidence that improved housing and biosecurity are critical in reducing pathogen incidence in herds.

**Supplementary Information:**

The online version contains supplementary material available at 10.1007/s00436-023-07797-4.

## Introduction

In Uganda, pig production has grown rapidly in recent years from approx. 0.7 million pigs in 1990 to 4.4 million in 2019 (UBOS 2020). This reflects a rise in the demand for pork (Ouma et al. 2014), which offers significant opportunities to pig producers for livelihood improvement. In Uganda’s current production systems, the lack of implementation of biosecurity measures constitute key factors for the spread of swine diseases such as African swine fever (Muhanguzi et al. 2012; Muhangi et al. 2014; Dione et al. 2016). Recent studies reveal that among diseases, respiratory and gastrointestinal (GIT) helminth infections are common in Ugandan pigs, contributing to the disease burden and thus affecting productivity in the sector (Ikwap et al. 2014; Roesel et al. 2017). In Lira district, Uganda, a recent multi-pathogen study revealed occurrence of *Mycoplasma hyopneumoniae (M. hyo)*, *Actinobacillus pleuropneumoniae (App)*, *Leptospira spp.*, porcine reproductive and respiratory syndrome virus (PPRSv), and porcine circovirus (PCV2) type 2 (Dione et al. 2018). Other studies confirmed presence of PCV2 in Ugandan pigs (Jonsson 2013; Ojok et al. 2013; Eneku et al. 2018). Three main production systems are identified: farrow to finish, farrow to wean, and wean to finish. In some farms, pigs are often not segregated by age groups and are fed together. Coupled with low biosecurity, this exposes younger pigs to infectious diseases. In Uganda, no pig vaccination was done against any pig disease during this study.

To date, no information exists on husbandry and management factors associated with important respiratory pathogen infections in Uganda’s smallholder pig systems. This hampers the design of effective interventions at farm level. Evidence from previous studies shows *Metastrongylu spp.* and *Ascaris spp.* compromise lung function due to the damage induced by their migratory larvae, thereby excerbating the effect of other viral and bacterial agents (Adedeji et al. 1989; Brewer and Greve 2011). This interaction increases disease duration and/or severity, with associated negative effects on productivity (Thacker et al. 1999). This study was designed to (i) identify risk factors for co-infections with respiratory pathogens, (ii) examine associations between gastrointestinal parasite infestations and key respiratory pathogens (PCV2, PRRSv, *M. hyo*, and *App*), and (iii) investigate associations between pathogens occurrence, farm management, and biosecurity practices, with a view to inform control and preventive measures at herd level.

## Materials and methods

### Study area

This study was done in Lira district, mid-northern Uganda, where the International Livestock Research Institute (ILRI) previously implemented a smallholder pig value chain development project (SPVCD) in 2011. In this project, a value chain assessment was conducted to select study sites using pig density, poverty levels, and market access (Ouma 2017). Our study used market access to select subcounties based on value chain domains into rural production for urban consumption (R-U) and urban production for urban (U-U) consumption (Ouma 2017). The total pig population in Lira district was estimated to be 30,000 in 2020 (district veterinary officer, personal communication). Pigs are produced under housed, tethered, and free-range systems (Kungu et al. 2019).Under these systems, pigs are housed in permanent or temporal structures made of cement, wood, or papyrus. Tethering is when pigs are tied on a rope (on a pole) to graze around the homestead, while free-range is when pigs are allowed to freely roam in the neighborhood in search of own feeds and water. Routine preventive measures such as anthelmintics are generally not practiced until pigs show visible signs of illness. In these smallholder pig production systems, biosecurity and hygiene practices are generally poor, which partly explains a high disease incidence in herds.

### Study design and sampling of subcounties, parishes, and villages

A cross-sectional serologic study was conducted from October to December 2018. We used multistage sampling to select subcounties and villages. In the first stage, four subcounties were selected (from a total of 9): two (central division and railways) representing U-U consumption and two (Adekokwok and Ngetta) representing R-U consumption. In stage two, two (2) villages with the highest pig density were selected for the study.

### Sample size determination

To determine the sample size, a formula for simple random sampling was used (Dohoo et al. 2003). A previous study in Lira district found a seroprevalence of *M. hyo* in pigs of 20.9% (Dione et al. 2018). Adjusting for test sensitivity and specificity, true prevalence was computed to be 24%. The required sample size of pigs was obtained from Eq. (1):

1 $${n=Z}_{\alpha /2}^{2}pq/{d}^{2}$$

where *n* = is the required sample size, *Z*_α_ is the standard *z*-score from a normal distribution (1.96), *p* = estimated prevalence of disease (24%), *q* = 1 − *p* (76%), and *d* = allowable error (6%). Using this formula, an unadjusted sample size of 195 pigs was computed. To adjust for within-farm clustering, we sampled 3 pigs per herd, thus the design effect (*Deff*) was obtained from Eq. 2 below:

2 $$\mathrm{Deff}=1+icc ({n}_{1}-1)$$

where icc is the intra-cluster correlation (0.2) for respiratory disease (Dohoo et al. 2003) and *n*_1_ is the number of pigs sampled per herd (3), thus the *Deff* calculated is 1.4. The adjusted sample size was calculated from the equation: *N* = *n*_1_ × number of pigs sampled per herd (3). From this, adjusted sample size of 273 pigs was derived.

### Sampling of farms and pigs

In each selected village, a list of pig keeping households/farms was obtained from the district veterinary office and the area local councils. Random sampling of farms was done until the required sample size was obtained. We sampled farms regardless of health status or anthelmintic treatments to examine differences in farm husbandry practices and how these influence occurrences of specific pathogens in farms. Using a sampling frame of all pig farmers generated with field research assistants, three (3) pigs per herd were sampled until the required sample size was reached. Only pigs ≥ 2.5 months old were selected for sampling, since pigs below that age are reported to retain maternal antibodies to PCV2 and PRRSv post weaning, which could interfere with serologic tests (Opriessnig et al. 2004; Gillespie et al. 2009). *App*-acquired colostral antibodies were reported to decay within 2 months postpartum (Vigre et al. 2003). We sampled pigs from 2.5 months and above regardless of health status, clinical signs, or feed types given.

### Data collection methods

A structured questionnaire with closed questions was designed, pre-tested by the first author in Mukono district, and revised before use. Research assistants were trained in its use before it was administered to each household head or farm manager. To ensure consistency, all questions were translated to a local language spoken in the area (Langi). The questionnaire captured data on potential risk factors for infection with respiratory pathogens.

### Blood sample collection and storage

Each pig was properly restrained as described in the ILRI Standard Operating Procedures (SOPs) manual, Sect. 2, parts (c) and (d) (ILRI 2004). Smaller pigs were restrained by hand while larger ones were restrained with a metallic pig catcher (model BZ002; MG. Livestock, Shandong, China) placed behind the upper incisor teeth and the snout raised upward. Blood was then collected from the cranial vena cava or jugular vein using a 21G, 1.5″ needle into plain 5-mL BD® vacutainer tubes. The tubes were labeled with animal identification details and then placed in an ice box at 4–6 °C. After collection, samples were delivered (within 3 h) to the district veterinary laboratory for temporary storage. Blood samples were left to stand at room temperature (20 °C) overnight and serum harvested the following day into 2 mL cryotubes (Sarstedt®, Germany), labeled, and stored in a fridge at − 20 °C until testing.

### Serological analysis

In the lab, sera were screened using ELISA assays according to manufacturers’ instructions for each pathogen: *M. hyo* and *App*-ApxIV (IDDEXX, Westbrook, Maine, USA) for PRRSv and PCV2 assays (Krishgen Biosystems, India). Cut-off sample to positive ratios (S/P%) for *M. hyo* were > 0.40 (positive) and < 0.30 (negative), *App* was ≥ 50% (positive) and < 40% (negative). PCV2 and PRRSv S/P cut-off ratios for positive and negative samples were ≥ 0.2 and < 0.2 respectively. Suspect samples were re-tested. Test sensitivity (*Se*) and specificity (*Sp*) for *M. hyo* ELISA were 85.6 and 99.6%, respectively; *Se* and *Sp* for *App*-ApxIV Ab ELISA test were 97.8 and 100%, respectively. *Se* and *Sp* for PRRSv were 94.0 and 94.0%, while for PCV2 *Se* and *Sp* these were 92.0 and 94.0%, respectively. Test *Se* and *Sp* were used to calculate true prevalence of respiratory infections at *α* = 0.05 significance level.

### Fecal sample collection and analysis

Fecal samples (~ 3 g) were collected from the rectum of each pig using gloved hands into 5-mL plastic containers, labeled, and placed in ice box at 4 °C. Samples were taken to the district veterinary lab for temporary storage at 4 °C. Samples were transferred to the Central Diagnostic Laboratory, College of Veterinary Medicine, Animal Resources and Biosecurity (CoVAB), Makerere University for analysis 1 week after collection. Helminth species were identified and fecal egg counts per gram (EPGs) were quantified using the Baermann and McMaster methods, respectively (MAFF 1986).

### Data analysis and presentation

Data was coded and entered into Excel 16.0, and any errors in entry were corrected by cross-checking with questionnaires. RStudio was used for data analysis and presentation (R Core Team 2019). True prevalence was computed by adjusting for apparent prevalence using prevalence 0.2.0 package in R, taking into account test sensitivities and specificities (Devleesschauwer et al. 2022). Multivariable logistic regression analysis of risk factors for each pathogen was performed. The response variable was the ELISA test result with predictors pig age and husbandry practices (house type, parasites, drug use, pig mixing, hygiene score, and drainage). The model below was fitted to predict respiratory infections as a function of pig characteristics and husbandry practices (house type, parasites, drug use, pig age, pig mixing, hygiene, and drainage):

3 $$\mathrm{ln}\frac{\widehat{p}}{(1-\widehat{p)}}={\beta }_{0}+ {\beta }_{1}{x}_{1i}+{\beta }_{2}{x}_{2i}\dots .....{\beta }_{p}{x}_{pi}$$

where $\mathrm{ln}\frac{\widehat{p}}{(1-\widehat{p)}}$ is the expected log of odds of infection, *β*_*0*_ is the model intercept, *β*_1_, *β*_2_ are regression coefficients, and *x*_1*i,*_* x*_2*i,*_ are the respective explanatory variables. Interaction terms were tested for each model, and confounding was checked by inclusion and exclusion of variables to observe a change in model coefficients. A cumulative link mixed-effects (CLMM) model was fitted to estimate the odds of co-infection (2 or more pathogens) with farm as a random effect. The CLMM model was fitted using R packages “factoextra” and “ordinal” using a backward elimination method such that all variables whose *p*-value > 0.2 were dropped. Residual plots and R-square statistics were used to assess the fitted models. Table 1 below presents housing and husbandry practice variables used in fitting the models.

**Table 1:** Housing and husbandry practice variables used in fitting the models

Variable category	Housing and husbandry practice variables	Level of measurement and codes
Housing	House type	0 = housed, 1 = tethered)
	Floor type	(2 = elevated platform, 1 = cement, 0 = soil/murram)
	Wall type	1 = bricks, 2 = mud/wattle, 3 = timber, 4 = wire mesh
	Roof type	2 = iron sheets, 1 = grass thatched, 0 = other material)
Biosecurity	Hygiene score	(0 = clean, 1 = moderate, 2 = poor/dirty)
	Frequency of pen cleaning	(0 = daily, 1 = 3–4 times/week, 2 = 1–2 times/week, 3 = never)
	Drug use	1 = yes: dewormers/antibiotics, 0 = none/other
	Wastes disposal	0 = none, 1 = thrown away, 2 = buried on ground)
	Drainage system	0 = none, 1 = sloping or pipe, 2 = sloping and pipe
	Isolate new/sick pigs	1 = no, 0 = yes
	Contacts with outside pigs	1 = no, 0 = yes
Pig level	Pig age	1 = 2–4 months, 2 = 5–8 months, 3 = > 8 months)
Respiratory pathogens	PRRSv	Binary; 1 = positive, 0 = negative
	PCV2	Binary; 1 = positive, 0 = negative
	*M. hyo*	Binary; 1 = positive, 0 = negative
	*App*	Binary; 1 = positive, 0 = negative
Parasites	*Strongyles spp*	Binary; 1 = positive, 0 = negative
	*Ascaris spp*	Binary; 1 = positive, 0 = negative

## Results

In all four subcounties, a total of 259 pigs were sampled from 90 farms. Data of 12 pigs was incomplete (missing values) and as such was excluded from the analysis. The median age category of the respondents was 36–50 years with an age range of 24–70 years. Of the 90 respondents, 41 (45.5%) were males and 49 (54.5%) were females. The median herd size per housed herds was 11 pigs, and for tethered herds it was 4 pigs. Male pigs constituted 53.7% (*n* = 139) of pigs in the sample while females were 46.3% (*n* = 120). The median age of sampled pigs was 5 months, and the age range was from 2.5 to 15 months. Table 2 below shows a summary of demographic characteristics.

**Table 2:** Demographic characteristics of respondents

Characteristic	Category	Males, *n* (%)	Females, *n* (%)	Total *n* (%)
Age (years)	18–35	16 (17.8)	21 (23.3)	37 (41.1)
	36–50	15 (16.7)	15 (16.7)	30 (33.3)
	51–75	10 (11.1)	13 (14.4)	23 (25.5)
Location/subcounty	Adekokwok (peri-urban)	18 (20.0)	29 (32.2)	47 (52.2)
	Central division (urban)	5 (5.5)	5 (5.5)	10 (11.0)
	Ngetta (rural)	9 (10.0)	9 (10.0)	18 (20.0)
	Railways div. (peri-urban)	9 (10.0)	6 (6.7)	15 (16.7)
Education level	None	1 (1.1)	2 (2.2)	3 (3.3)
	Primary	10 (11.1)	31 (34.4)	41 (45.5)
	Secondary	15 (16.7)	7 (7.8)	22 (24.5)
	Tertiary	15 (16.7)	9 (10.0)	24 (24.7)
Occupation	Farmer	29 (32.2)	36 (40.0)	65 (72.2)
	Business	7 (7.8)	8 (8.9)	15 (16.7)
	Employee	3 (3.3)	4 (4.4)	7 (7.7)
	Others	2 (2.2)	1 (1.1)	3 (3.3)
Management system	Housed	26 (28.9)	28 (31.1)	54 (60.0)
	Tethered	15 (16.7)	21 (23.3)	36 (40.0)
Breeds kept	Local	4 (4.4)	4 (4.4)	8 (8.8)
	Exotic	7 (7.8)	5 (5.5)	12 (13.3)
	Cross-bred	28 (31.1)	36 (40.0)	64 (71.1)
	Mixed	2 (2.2)	4 (4.4)	6 (6.6)
Totals		41 (45.5)	49 (54.5)	90 (100.0)

### Prevalence of respiratory pathogens and GIT parasites

Table 3 below shows true herd and individual level prevalence of selected respiratory pathogens at respective 95% confidence intervals. Results showed that *App* was the most prevalent pathogen while PCV2 was the least prevalent at both pig and herd levels. Of the GIT parasites, *Eimeria spp* was the most prevalent while *Trichuris spp* was the least prevalent parasite found.

**Table 3:** True prevalence of tested respiratory pathogens and helminths in pigs

Pathogen	Individual pig level (*n* = 259)		Herd level (*n* = 90)	
	% Prevalence	95% Conf. int	% Prevalence	95% Conf. int
PCV2	3.50	0.4–7.7	23.9	15.6–33.1
PRRSv	14.0	9.1–19.5	32.6	23.4–42.6
*M. hyo*	6.8	3.8–10.6	16.2	9.5–24.6
*App*	30.5	25.0–36.2	45.7	35.6–55.9
*Ascaris spp*	12.7	9.2–17.3	28.9	20.5–38.9
*Strongyles spp*	16.2	12.2–21.2	34.4	25.4–44.7
*Trichuris spp*	1.5	0.6–3.9	4.4	1.7–10.7
*Eimeria spp*	56.4	50.3–62.3	81.1	71.8–87.8

Conf. int, confidence interval

### Prevalence and proportion of co-infections

Co-infections with two or more pathogens were observed in this study. Among respiratory pathogens, the highest percentage of co-infections was between pathogens and GI parasites. Among pathogens, the highest co-infections occurred between PRRSv and PCV2, followed by *M. hyo* and PCV2. Only 5 pigs were co-infected with 3 pathogens, and 42.5% (*n* = 110) of pigs sampled had at least 2 co-infections. Table 4 below shows a summary of percentage of pigs co-infected with pathogens and GI parasites.

**Table 4:** Percentage of pigs with respiratory co-infections and concurrent GI helminths

Pathogen species	Number and percent of pigs with co-infections			
	PCV2 (*n* = 9)	PRRSv (*n* = 36)	*M. hyo* (*n* = 18)	*App* (*n* = 79)
PCV2	-	5 (13.8%)	3 (16.7%)	9 (11.4%)
PRRSv	5 (55.5%)	-	4 (22.2%)	15 (18.9%)
*M. hyo*	3 (33.3%)	4 (11.1%)	-	4 (5.0%)
*App*	9 (11.4%)	15 (41.6%)	4 (22.2%)	-
*Ascaris spp*	8 (89%)	9 (25%)	9 (50%)	14 (17.7%)
*Strongyles spp*	6 (66.7%)	8 (22.2%)	8 (44.4%)	16 (20.2%)
*Eimeria spp*	5 (55.5%)	31 (86.1%)	10 (55.6%)	55 (69.6%)

### Multivariable logistic regression model of risk factors for individual respiratory infections

Results showed that parasite infections, pig confinement (only for *App*), drug use, and pig age were significant predictors of single respiratory infections (Table 5). Drugs used by farmers were antibiotics, anthelmintics, and multivitamins.

**Table 5:** Multivariable logistic regression models of risk factors for individual respiratory infections

Pathogen	Variable	Coeff	Std error	OR	95% CI	*z*-value	*p*-value
PCV2	(Intercept)	− 2.4358	0.3214	0.087	0.044—0.157	− 7.578	< 0.001***
	*Ascaris spp.* infec	1.5590	0.4949	4.753	1.738–12.38	3.150	0.001**
	No pig mixing	− 0.690	0.485	0.501	0.182–1.260	− 1.421	0.155
PRRSv	(Intercept)	− 2.530	0.726	0.079	0.017—0.307	− 3.485	< 0.001***
	Pig age	0.181	0.069	1.199	1.046–1.378	2.614	0.008**
	Herd size (> 20 pigs)	0.016	0.005	1.016	1.005–1.028	2.935	0.003**
	Hygiene score_1	1.003	0.584	2.728	0.953–9.949	1.716	0.086
	Hygiene score_2	0.030	0.775	1.031	0.216–4.938	0.039	0.968
	Drug use	− 2.125	0.669	0.119	0.028–0.406	− 3.176	0.001**
	Farmer sex (fem.)	− 1.207	0.430	0.299	0.126–0.692	− 2.803	0.005**
	Drug use*farmer sex (female)	2.021	0.937	7.550	1.116–47.929	2.157	0.031*
*M. hyo*	(Intercept)	− 3.5154	0.4771	0.0297	0.010–0.068	− 7.369	< 0.001***
	*Strongyles spp*	2.5628	0.5824	12.971	4.300–44.171	4.401	< 0.000***
	Drainage	− 0.7957	0.6885	0.4512	0.096–1.570	− 1.156	0.248
*App*	(Intercept)	− 2.881	0.50961	0.056	0.019—0.145	− 5.654	< 0.001***
	Housed	1.138	0.358	3.122	1.591–6.541	3.178	0.001**
	Pig age > 6 months	0.12313	0.055	1.131	1.014–1.263	2.205	0.027*
	*Eimeria spp*	0.793	0.303	2.212	1.232—4.061	2.620	0.008**

Drugs used by farmers: antibiotics, anthelmintics, and multivitamins. Hygiene score_0 = clean, hygiene score_1 = moderate, hygiene score_2 = poor. Hygiene score was made by physical observation of the level of hygiene in pens during sampling. * = *p* < 0.05, ** = *p* < 0.01, and *** = *p* < 0.001

### A cumulative link mixed model for mixed respiratory infections with farm as random effect

Table 6 below shows a cumulative link mixed model (CLMM) from R packages (“ordinal,factoextra”) of factors for respiratory co-infections with farm as a random effect. This model was fitted to predict co-infections (regardless of pathogens involved), as these have synergistic effects on the induction of respiratory disease. The model showed that farmer occupation, floor type (cement, elevated floor), and no contacts with outside pigs were protective against co-infections, while pig age, wall type (mud, timber), herd size, and helminth infestations increased risks of respiratory co-infections. A likelihood ratio test (LR stat) statistic (4.02, *p* < 0.04*) showed that the model below was slightly significant.

**Table 6:** A cumulative link mixed model for respiratory co-infections in pigs

Independent Variable	Estimate	Std. error	OR	95% CI	*z*-value	*p*-value
Thresholds 0|1	3.02	0.69	20.62	5.26 − 8.08e + 01	4.34	-
1|2	5.55	0.77	257.54	56.80 − 1.16e + 03	7.19	-
2|3	7.68	0.91	2185.51	366.93 − 1.30e + 04	8.44	-
Occupation_2	− 1.14	0.40	0.32	0.14 − 7.08e − 01	− 2.81	0.004**
Occupation_3	− 1.82	0.69	0.16	0.04 − 6.27e − 01	− 2.63	0.008**
Occupation_4	− 0.38	0.79	0.68	0.14 − 3.20e + 00	− 0.48	0.626
Pig age	0.20	0.05	1.23	1.10 − 1.38e + 00	3.57	0.0003***
Herd size	0.01	0.00	1.01	1.00 − 1.02e + 00	2.95	0.003**
Floor type cement	− 1.14	0.45	0.31	0.13 − 7.79e − 01	− 2.50	0.012*
Floor type raised	− 1.51	0.65	0.22	0.06 − 7.95e − 01	− 2.31	0.020*
Wall type 1 (mud)	2.88	0.81	17.91	3.66 − 8.76e + 01	3.56	0.0003***
Wall type 2 (timber)	1.10	0.56	3.03	1.00 − 9.13e + 00	1.97	0.048*
Wall type 3 (bricks)	0.35	0.48	1.42	0.55 − 3.66e + 00	0.73	0.468
Hygiene score 1	0.83	0.46	2.31	0.93 − 5.73e + 00	1.80	0.070
Hygiene score 2	0.96	0.59	2.62	0.81 − 8.48e + 00	1.61	0.106
Cleaning frequency 1	0.83	0.50	2.30	0.85 − 1.30e + 04	1.65	0.099
Cleaning frequency 2	1.88	0.48	6.58	2.54 − 1.70e + 01	3.89	0.000***
Cleaning frequency 3	1.46	0.44	4.32	1.80 − 1.04e + 01	3.27	0.001**
No contacts	− 0.78	0.45125	0.45	0.18 − 1.10e + 00	− 1.73	0.082
*Strongyles spp*	1.25	0.37402	3.50	1.68 − 7.29e + 00	3.35	0.0008***
*Ascaris spp*	1.23	0.42588	3.45	1.49 − 7.95e + 00	2.91	0.003**

For categorical independent variables, higher or better was given with higher codes except for hygiene/cleaning frequency; contacts with outside pigs was coded as 1 = no and 0 = yes); pig age (months) and herd size were entered as counts. Potential collinearity between “cleaning frequency” and “hygiene score” was tested and found to be low (0.12) before both were included in the model. Differences in practices between farms account for the random effect. OR, odds ratio

* = *p* 0.05, ** = *p* < 0.01, and *** = *p* < 0.001

## Discussion

These results highlight widespread occurrence of selected respiratory pathogens in pigs in the study area. At both individual and herd levels, *App* was found to be of highest prevalence, followed by PRRSv, PCV2, and, lastly, *M. hyo* (Table 3). These findings are comparable with those from a recent study (Dione et al. 2018). However, compared to the findings of other studies (Jonsson 2013; Ojok et al. 2013; Eneku et al. 2018), our study found a lower PCV2 seroprevalence. This may be due to differences in the sampling procedures, diagnostic methods used, and the type of production system from which pigs were sampled. In our study, sampling was done in households that confined their pigs in pig sheds or tethered around homesteads, while Jonsson (2013) sampled pigs from a wildlife-livestock interface (near Murchison Falls national park), which probably exposed them to a higher risk of infection from other roaming or wild pigs. Eneku et al. (2018) sampled pigs that presented with clinical signs of PCV2 and therefore had a higher probability of PCV2 detection while another research team (Ojok et al. 2013) sampled pig tissues from a local abattoir.

The PCV2 seroprevalence at individual pig and herd levels found in this study was lower than (54 and 78% respectively) that reported in Mozambique (Laisse et al. 2018). Differences in the sampled population could account for variations in PCV2 prevalence, as the Mozambiqan study was done in slaughter places and was likely on older animals compared to the pigs sampled in this study. The results from this study also show that PCV2 seroprevalence was higher (6.9% vs 1.4%) than that found in Nigeria (Aiki-Raji et al. 2018) and lower than (15.9%) that found in South Africa (Afolabi et al. 2017).

This study revealed a higher PRRSv seroprevalence compared to the previous findings (Dione et al. 2018). This could suggest either increased herd to herd transmission over the past few years, since PRRSv transmission can occur via several routes (Otake et al. 2010), or because the virus can remain in affected herds as a persistent infection (Murtaugh and Genzow 2011). The finding that the odds of testing seropositive to PPRSv rises with increase in pig age is in consonance with findings from previous studies that showed that neutralizing and anti-PRRSv IgG antibodies can remain persistent for several months (Nelson et al. 1994; Murtaugh et al. 2002). The increased odds of seropositivity to PRRSv due to lack of regular preventive treatments against bacterial and helminth infections were demonstrated in this study. The role of PRRSv in inducing severe disease during co-infections with other pathogens has been previously reported (Halbur et al. 1996; Thacker et al. 1999). This suggests that regular prophylactic treatments are important in reducing the risk of opportunistic co-infections. A similar observation was made for *App*, in which infection was dose-dependent, accounting for increased incidence in older pigs (Marsteller and Fenwick 1999). The effect of herd size was highlighted in this study. The observation that larger herds (> 20 pigs) increased the odds of PRRSv infection may be related with increased stocking density, as PRRSv is known to be highly infectious. The increase in the odds of PRRSv detection may also be due to its tendency to remain as a persistent infection after entry into a herd (Pileri and Mateu 2016).

Co-infections in this study were lower than in other studies (Gillespie et al. 2009). Co-infections between PRRSv and *App* were the most prevalent, followed by PCV2 and *App*. The effect of PCV2 co-infection with other pathogens in increasing the severity and incidence of PCV2-associated disease has been reported in previous studies (Opriessnig et al. 2004; Fablet et al. 2012; Segalés et al. 2013). Other studies reveal that a diversity of pathogens is involved in respiratory disease (Qin et al. 2018). The cumulative link model showed that better floor types (cement or elevated) had protective effects against co-infections (Table 6).While hygiene score was not significantly associated with co-infections, the frequency of cleaning of pens was significantly associated with co-infections: the poorer the level of hygiene and the lower the frequency of cleaning of pens, the higher the risk of co-infections. These findings concur with previous studies which showed pigs raised in clean environments grew significantly faster than those raised on dirty floors (Cargill 2019).

This study revealed associations of particular respiratory pathogens and GIT parasite infections in pigs (Tables 4 and 5). These results are comparable with findings from a study in southwest Uganda which reported a high prevalence of GIT helminth infections (Roesel et al. 2017). The increased odds of *Ascaris spp* infection in tethered pigs illustrates the importance of biosecurity (e.g., confining pigs) in reducing the risk of infection. *Ascaris spp.* has been shown to compromise lung function through immunomodulatory mechanisms*,* thereby excerbating the effect of other viral and bacterial agents, as well as increase disease severity (Adedeji et al. 1989; Brewer and Greve 2011). While pigs infected with *Eimeria spp.* may show no observable clinical signs, *Eimeria spp.* has been reported to cause diarrhea in piglets as it damages intestinal mucosa, increasing susceptibility to other pathogens.

In general, results showed that biosecurity variables had a significant influence on pathogen occurrence in farms. These findings agree with previous studies which revealed that good housing, hygiene and reduced stress play a significant role in minimizing the effects of diseases such as PCVAD (Gillespie et al. 2009; Cargill 2019). Poor drainage and hygiene in some farms may have raised the risk of re-infections with parasite eggs from contaminated feeds and water. In addition, the higher parasite infestations observed in some farms (tethering) may also have been due to lack of implementation of routine preventive measures such as deworming. These environmental stressors (poor hygiene, rearing of different age groups in the same pens, overcrowding, poor nutrition, etc.) are known to suppress immunological responses and therefore impede a pig’s ability to fight off infection (Cargill 2019). The role of good ventilation and proper cleaning practices in improving indoor air quality by reducing microbial contamination with respiratory pathogens has been documented (Banhazi et al. 2008; Cargill 2019). Overall, the results of the logistic regression models demonstrated the importance of improved hygiene and biosecurity in reducing mixed respiratory infections.

## Conclusions

This study highlights widespread occurrence of economically important respiratory pathogens in pigs in the study area. This may likely reflect the situation in swine herds in eastern and northern Uganda, where production systems are largely similar. The negative correlations between biosecurity variables and mixed pathogens signify the role of improved biosecurity in reducing the risks of co-infections. In addition, the associations between biosecurity and housing and pathogens provides further support to the above evidence. Further studies to identify PCV2 and PRRSv genotypes that circulate in pigs in this region, as well as quantify their economic impacts on swine productivity, are warranted to guide the design of effective interventions.

## Limitations of the study

Statistical analyses were limited to only estimates of prevalence and odds of infections. Potential bias may have been introduced by inaccurate responses to some questions in the questionnaire and misclassification of pig errors due to imperfect assay sensitivities and specificities. Also, the time lapse between fecal sample collection and lab analysis may have affected the sensitivity of the Baerman’s test, as ability of larvae to hatch was reduced. The detection of antibodies may not reflect actual or current infection. This may have led to over- or underestimation of the observed relationships.

## Supplementary Information

Below is the link to the electronic supplementary material.

Supplementary file1 (DOCX 34 KB)

## Data Availability

The original contributions presented in the study are publicly available. The dataset analyzed for this study can be found here: https://data.ilri.org/portal/dataset/multipathogen-survey-and-risk-factors.
