# Supplementary material for: Co-infections of respiratory pathogens and gastrointestinal parasites in smallholder pig production systems in Uganda
Source: Parasitol Res. 2023 Feb 22;122(4):953–62. doi: 10.1007/s00436-023-07797-4 (PMC10006049; doi:10.1007/s00436-023-07797-4)
Supplement: Supplementary file 1 — Supplementary file1 (DOCX 34 KB) [file 436_2023_7797_MOESM1_ESM.docx]

***HH Questionnaire HH no…………………..***

**Prevalence and risk factors of respiratory infections in Ugandan pigs**

Dear Respondent,

This study aims at identifying important respiratory pathogens and risk factors for their occurrence in pigs in Uganda. The information generated will be used to advise you and other farmers on prevention and control measures.

As a key actor in this sector, you are requested to take part in this study. The information collected here remains confidential and will be used purely for research purposes only. We shall share the findings with you at the end of the study, which would help you improve the productivity and profitability of your piggery enterprise.

Thanks for your participation.

…………………………………….

Peter Oba *(PhD Student, ILRI/Makerere University)*

***Mobile: 0772 694099/ 0700 474633***

**PART A: GENERAL INFORMATION**

Date of interview:……………………………. (i) District …………………………. (ii) Sub county: …………………………………….

(iii) Parish:…………………………… (iv) Village:……………………………………. (v) GPS reading:………………………………

(vi) Name of respondent (optional):……………………………………… (vii) Sex: 1= Male 2= Female

(viii) Age category: 1= 18-35 years

2= 36-50 years

3= 51-75 years

4= 76 and above

(viii) Education level: 1= Primary 2= Secondary 3= Tertiary 4= Post-graduate

(ix) Religion: 1= Catholic 2= Protestant 3= Muslim 4= Pentecostal 5= Others

(x) Main occupation: 1 = Farmer 2 = Business 3 = Formal employee 4=other (specify)…

**PART B: HERD SPECIFIC INFORMATION**

1.1 Production system/type

1 = Intensive

2 = Semi-intensive

**1.2 Herd structure**

1.2.1 Breed of pigs kept: 1= Local 2 = Exotic 3 = Crossbreed 4 = Mixed (exotic and local)

1.2.2 If cross breed, specify breed:……………………………

| Age group | Breed | Number | Age (months) |
| --- | --- | --- | --- |
| Sows |  |  |  |
| Gilts |  |  |  |
| Growers/fatteners |  |  |  |
| Weaners |  |  |  |
| Piglets |  |  |  |
| Boars |  |  |  |
| Total |  |  |  |

**2.0 HERD ENVIRONMENT**

2.1 **Housing system**

2.1.1 Housing structures 1 = housed

0 = not housed (tethered)

2.1.2 If housed:

Specify floor type: 2 = raised (with wood / timber above the ground)

1 = not raised (concrete floor)

0 =not raised, soil or murram

2.1.2 If not raised, specify type of floor

| Type of floor | Est. size (m^2^) | Comments |
| --- | --- | --- |
| Concrete floor |  |  |
| No concrete floor (rammed earth or murram) |  |  |
| Timber boards |  |  |

2.1.3 Wall material: 4 = Bricks

3 = Mud and wattle

2 = Timber boards

1 = Wire mesh

2.1.4 Roofing material 2 = iron sheets

1 = grass thatched / papyrus

0 = other (specify)…………………………………………………….

2.2 **Stocking density** (0 = >1 pig per sq. metre, 1 = 1 or less pig per sq. metre)

| Pig age category | Number of pigs | Approx. area of pen (m^2^) | Density (pigs/m^2^) |
| --- | --- | --- | --- |
| Sows |  |  |  |
| Gilts |  |  |  |
| Growers/fatteners |  |  |  |
| Weaners |  |  |  |
| Piglets |  |  |  |
| Boars |  |  |  |

**2.4 Feeding:** Feed types given to pigs (you can choose more than one)

| Feed type used | Est. qty per day (kg) | No. of times pigs are fed per day |
| --- | --- | --- |
| Household leftovers |  |  |
| Local mixed feed |  |  |
| Green forages |  |  |
| Maize bran |  |  |
| Commercial feeds |  |  |

**PART C: HERD HEALTH PERFORMANCE**

3.1 What disease control and preventive measures do you use ?

1= dewormers/antibiotics

0= do nothing)

Frequency of practice: 1 = ≥ 1 time /every 3 months

0 = ≤ 1 time/every 3 months

3.2 **Preventive drugs used (in the last three months)**

**3.4 In what circumstances do you use the drug(s) named above?**

**Frequency:** 1= routine (1-2 times per month); 2= occasional (once every 3-4 months)

3 = rarely (once every 5-6 months)

| Type of drug | Frequency | Reason for use | Comments |
| --- | --- | --- | --- |
|  |  |  |  |
|  |  |  |  |
|  |  |  |  |

**Reasons for use:** 1= to keep pigs healthy

2 = only when pigs show signs of illness

3 = other (specify)….

**3.5 Important signs of respiratory disease(s) observed in the last one month (you may tick more than one):**

Yes = 1 No = 0

| **Code** | **Sign** | **Yes** | **No** |
| --- | --- | --- | --- |
| 1 | Sneezing |  |  |
| 2 | Coughing |  |  |
| 3 | Difficult / labored breathing |  |  |
| 4 | Fever (high temperature) |  |  |
| 5 | Loss of appetite |  |  |
| 6 | Sudden death |  |  |
| 7 | Diarrhea |  |  |
| 8 | Other (specify)…… |  |  |

**3.6 Age group most affected by respiratory signs** (enter appropriate code)

| Age group | Not affected | Slightly affected | Moderately affected | Highly affected |
| --- | --- | --- | --- | --- |
|  | 0 | 1 | 2 | 3 |
| Piglets |  |  |  |  |
| Weaners |  |  |  |  |
| Growers / fatteners |  |  |  |  |
| Adults |  |  |  |  |

3.7 Period when most signs are observed

1= rainy season

0 = dry season

3.8 When you see signs of respiratory disease, what do you do?

1= call a vet / extension worker

2 = buy drugs from local shop and administer myself

3= get local herbs and administer

4 = sell the pig(s)

5= do nothing

6 = other…………..

3.9 Do you isolate sick pigs from healthy ones when you notice them?

1= yes

2 = no

**PART E: HERD MANAGEMENT:** *this section aims at understanding the general herd management practices that shall be used to identify possible risk factors for occurrence of respiratory disease*

**4.1 Biosecurity on the farm:**

1= Fenced

0= No fence

**4.2 Disinfectant use**: 1= Disinfectant used

0= No disinfectant used

**4.6 Labour:** Who takes care of or feeds the pigs?

1= Husband 2= Wife 3= Household member/child 4= Casual laborer (hired)

If own or hired labour, estimated cost per person per month (UShs)…………………………….

4.7 Do you mix pigs of different age groups in the same pen/unit (apart from a lactating sow)?

1= no

0= yes

4.9 **Hygiene and sanitation:**

4.9.0 Hygiene score

2= dirty/filthy

1=moderate

0= clean

4.9.1 Cleaning frequency per week

0= daily

1= 3-4 times/week

2= 1-2 times/week

3= Never

4.9.2 What is the type of drainage used in the pig houses?

0= None

1= sloping or pipe

2= sloping & pipe

4.9.3 Where do you dump pig wastes?

1 = > 10 m away from the pens

0 = < 10 m away from pens

4.9.4 How do you dispose of pig fecal material?

0=None

1=Thrown away

2=Buried on ground

**5.0 Visitors to the farm**

How often do visitors or other people enter your farm (piggery unit)?

0= 'daily' or 4-5 times /week,

1= 'occasionally' (1-2 times /week),

2= rarely/never

5.1 How far or near are other pig farms from yours?

1 = < 20 m

2 = between 20 - 50 m

3 = more than 50 m

5.2 Do pigs from neighboring farms often mix with your pigs?

1 = no

0 = yes

5.3 How do you market or sell your pigs?

1 = take to the local market

2 = bring a trader to buy them from home

3 = slaughter in a nearby slaughter slab

4 = other (specify)………………………………….

**PART F: HERD BREEDING MANAGEMENT**

6.1 What breeding system do you use at your farm? 1 = natural service 2 = artificial insemination (AI)

6.2 If by natural service, which boar do you use?

0= borrowed from neighbor,

1= owned

6.3 If borrowed from a neighbor, how is the boar moved?

1= brought up to home 2 = a sow is taken where the boar is.

6.4 Do you buy replacement stock from outside your farm? 1= yes 2 = no

6.5 If yes, how often (in six months’ time)? …………………………………..

6.6 If yes, which age of pigs do you buy or obtain as replacement stock? (you may tick more than one)

1= adults

2 = gilts

3= weaners/growers

4 = piglets

5 = mixed age groups

6.7 Do you isolate new or sick pigs (before mixing with your pigs) brought to your farm?

1= yes

0 = no

Any other comments you wish to state………………………………………………………………………………………………

………………………………………………………………………………………………………………

*End of questionnaire and thank you for your participation*
